# A Trans-Theoretical Approach to Physical Activity Profile in General Population of Mashhad

**DOI:** 10.5539/gjhs.v7n7p46

**Published:** 2015-03-26

**Authors:** Zahra Abbasi Shaye, Mojtaba Mousavi Bazzaz, Veda Vakili

**Affiliations:** 1 School of Medicine, Mashhad University of Medical Sciences, Department of Community Medicine, Mashhad, Iran

## Abstract

Regular physical-activity is necessity for a healthy lifestyle. Despite public health efforts, a minority of population are involved in healthy levels of physical-activity. This study provides evidence about exercise patterns and predictors of Mashhad-Iran population according to TTM change stages. In this cross-sectional study, we surveyed a total number of 564 participants from Mashhad in 2014 by using stages of change questionnaire. Analysis showed 23.4% of participants were in pre-contemplation stage, 18 in contemplation, 24.6% in preparation, 8.10% in action, 14.4% in maintenance and 11.5% were in termination phase. Age, gender, BMI, alcohol consumption, sleep duration, having compeer and encouragement were identified as predictors of pre-contemplation stage. Genders, having company and using bicycle for transportation were predictors of termination phase. Tailor interventions based on the predictors to enhance the physical activity among specific subgroups would be of interest.

## 1. Introduction

Regular physical activity enhances public health status and contributes to prevention of chronic diseases. In particular, obesity, type II diabetes mellitus, coronary heart disease, atherosclerosis, hypertension, osteoporosis, and various kinds of cancers can be prevented by appropriate physical activity (WHO, 2010; Rehn, Winett, Wisløff, & Rognmo, 2013; Kruk & Aboul-Enein, 2007; Li & & Siegrist, 2013). Physical activity can significantly decrease overall mortality rate in general population, since a large number of deaths occurred each year are due to inactivity (Lee & Skerrett, 2001; Wen, Wai, & Tsai, 2011).

In 2008, United States Department of Health and Human Services developed the physical activity guideline, recommending adults with 18 or more years of age to have at least 150 minutes of moderate-intensity (5 periods in a week, each last for at least 30 minutes) or 75 minutes of vigorous-intensity physical activity in a week (Committee PAGA, 2008). However, many studies have indicated that the majority of society in developed countries does not adhere to the recommended guidelines regarding effective and beneficial physical activity (Craig, Mindell, & Hirani, 2008; Pleis & Lucas, 2009). This lack of physical activity seems to be more prevalent in Iranian population, since approximately 80% of Iranians are not sufficiently active (Sheikholeslam, Mohamad, Mohammad, & Vaseghi, 2004).

An evidence based underlying mechanism for this sedentary lifestyle is behavioral stages of changes. Transtheoretical model (TTM) is a framework, which categorizes people concerning their readiness and willingness to change. TTM introduces six stages of change, including precontemplation, contemplation, preparation, action, maintenance, and termination. TTM successfully addressed the concerns over exercise behavior changes in different populations and brought interventional strategies for each stage (Prochaska & Velicer, 1997). The change process in physical activity behavior is linked to individuals and environmental interactions. Researchers suggest that behavioral processes are utilized to intervene in later stages and as the stage promote self-efficacy of individuals increases (Rhodes, Plotnikoff, & Courneya, 2008; Mori et al., 2009).

The aim of the present study was to apply the TTM on general population of Mashhad city and evaluate the change levels and possible relative factors regarding physical activity.

## 2. Methods

In this cross-sectional study, we surveyed a total number of 564 participants from Mashhad, Iran in 2014. Mashhad is the second populous city in Iran and is the capital of Razavi Khorasan Province. It is located in the north east of the country close to the borders of Afghanistan and Turkmenistan. Its population was 2,772,287 at the 2011 population census. Housing the holy shrine of eighth Shia Imam, Mashhad receives millions of pilgrims each year. For data collection we referred to public transport stations, public parking lots, car parks of shopping centers, banks, hospitals and universities all around the city. Parking of the holy shrine was also a place for sampling collection procedure.

Survey was done using a checklist and stages of change questionnaire (Marcus, Selby, Niaura, & Rossi, 1992). The checklist included the socio-demographic characteristics and possible related factors of physical activity behavior. The questionnaire consisted of six questions with yes and no answers, according to six stages of change. Stages of change refer to a person’s readiness to engage in regular exercise. Someone in pre-contemplation (pc) does not exercise and is not planning to start exercising within 6 months. A contemplator does not exercise but is planning to start within 6 months. A person in preparation is planning to start exercising within 1 month and has taken some initial steps toward it. Someone in action has been exercising for less than 6 months. A person in maintenance has been exercising for 6 months or more and finally the person in termination stage, will never leave exercising (Reynolds, Spruijt-Mtz, & Unger, 2008).

We used Persian translated version of the questionnaire, being valid and reliable before (Moattari, Shafakhah, & Sabet Sarvestani, 2013). Demographic information, including age, sex, education level, job status, history of smoking and drug or alcohol abuse were asked in the checklist. A total number of 564 questionnaires were completed.

Ethics Committee of Mashhad University of Medical Sciences approved the study. The interviewers explained the objectives of research for participants and assured them about the privacy of their personal data and after getting the consent, they filled the questionnaires.

SPSS 11.5 software (SPSS Inc., Chicago, Illinois, USA) was used for all statistical analyses. Standard descriptive statistics were applied to describe the pattern of the data. Chi-square test was used to examine the significance of the association between categorical data. Normality of the data was checked with Kolmogorov–Smirnov test. ANOVA and Kruskal-Wallis tests were applied in normal and non-normal distributions respectively. Logistic regressions were used to predict the factors’ influence on marriage instability. All tests were 2-tailed, and probability values 0.05 were considered significant.

## 3. Result

We had 564 participants in our research. The data showed that 316 (56.3%) of participants were male and 245 (43.7%) were female. Average age of the participants was 28 years with maximum of 84 and minimum of 11 years. 256 (45.5%) of participants were single; among them 19 (3.4%) were divorced and seven (1.2%) were widowers, 306 (54.5%) of participants were married. Among the respondents 136 (26.8%) of them were jobless/housekeeper, 233 (45.9%) were employed and 139 (27.4%) were students. 3 participants (0.5%) were illiterate, 211 (37.9%) had nonacademic education and 343 (61.6%) had academic education. Frequency distribution of participant’s demographic characteristics and factors related to respondent’s physical activity are shown in Table 1.

**Table 1:** Demographic Characteristics of study participants

		Total	Man	Woman
**Family size**		4(0-12)	4 (0-12)	3 (1-10)
**Distance to nearest park (meter)**		375(1-10000)	500 (1-10000)	200 (2-10000)
**Distance to nearest** gym (meter)		500(3-20000)	500 (3-20000)	250 (4-17000)
**Compeer**		238(43)	144 (46.2)	91 (38.1)
encouraging		322(59.8)	184 (59.4)	145 (59.9)
Fast food / processed food (at month)		3(0-50)	3 (0-50)	2 (0-30)
Useless Junk (at month)		2(0-100)	2 (0-500)	2 (0-100)
Sitting position (hours a day)		7(1-20)	7 (1-20)	8 (1-16)
Sleep duration (hours a day)		8(2-14)	7(2-14)	8 (2-14)
Transportation mode	Personal car	277(49.1)	154 (48.1)	122 (49.8)
	taxi	89(15.8)	35 (11.1)	53 (21.6)
	bus	153(27.1)	89 (28.2)	62 (25.3)
	metro	97(17.2)	54 (17.1)	42 (17.1)
	bicycle	9(1.6)	8 (2.5)	0 (0)
	pedestrian	69(12.2)	44 (13.9)	24 (9.8)
BMI (kg/m^2^)		23.84 (14.69-45.21)	23.87 (14.69-45.21)	23.51 (15.62-44.37)
Past medical history	diabetes	20(3.5)	12 (3.8)	8 (3.3)
	CVD	27(4.8)	18 (5.7)	9 (3.7)
	Hepatobiliary system	12(2.1)	8 (2.5)	4 (1.6)
	respiratory	15(2.7)	10 (3.2)	5 (2)
	cancer	5(0.9)	1 (0.3)	4 (1.6)
smoking(pack/year)		107(19.1)	3 (0.1-72000)	1.56 (2-480)
Hookah smoking		101(17.9)	70 (22.2)	31 (16.7)
alcohol		59(10.5)	43 (13.6)	16 (6.5)
addiction		15(2.66)	11 (3.5)	4 (1.6)

Analysis showed that 124 (23.4%) of participants were in pre contemplation stage, 95 (18%) were in contemplation, 130 (24.6%) were in preparation, 43 (8.10%) were in action, 76 (14.4%) were in maintenance and 61 (11.5%) were in termination phase. Frequency of individuals in each stage separated by gender is shown in Table 2 which indicates a statistically significant difference between men and women in this frequency (p-value<0.05).

**Table 2:** Frequency distribution of participants according to stages of change

	Men N (%)	Women N (%)	p-value	Total N (%)
PC	77 (25.8)	46 (20.2)		123 (23.4)
C	54 (18.1)	40 (17.5)		94 (17.9)
P	54 (18.1)	75 (32.9)		129 (24.5)
sedentary citizens	185 (35.2)	161 (30.6)	0.003	346 (65.8)
A	26 (8.7)	17 (7.5)		43 (8.2)
M	44 (14.8)	32 (14)		76 (14.4)
T	43 (14.4)	18 (7.9)		61 (11.6)
Active citizens	113 (21.5)	67 (12.7)		180 (34.2)

Demographic characteristics and several related factors to physical activity are listed in Table 3 by different stages of trans-theoretical model in exercise patterns. Our analysis showed that average age, sleep duration, hours of sitting position, distance to nearest sport club (meter) and alcohol consumption were significantly different between stages of change for exercising(p-value < 0.05).

**Table 3:** Demographic characteristics for exercise patterns according to stages of change of TTM model

		PC	C	P	A	M	T	P-Value
**Age: median(min-max)**		28(16-67)	30(15-84)	26.5(14-70)	27(12-65)	33(15-65)	26(18-70)	0.002
**Family size**		3(1-7)	4(0-12)	4(1-9)	4(1-11)	4(1-10)	4(1-12)	0.990
**Marital status (N (%))**	Single	56(22.8)	47(19.1)	62(25.2)	22(8.9)	30(12.2)	29(11.8)	0.770
	married	67(23.8)	48(17.1)	68(24.2)	20(7.1)	46(6.4)	32(11.4)	
**Education (N (%))**	Non academic	54(28.1)	36(18.8)	41(21.4)	15(7.8)	23(12)	23(12)	0.320
	academic	68(20.6)	58(17.6)	88(26.7)	26(7.9)	53(16.1)	37(11.2)	
**Smoking (N (%))**	yes	32(30.8)	20(19.2)	23(22.1)	9(8.7)	13(12.5)	7(6.7)	0.270
	no	91(21.6)	75(17.8)	107(25.4)	33(7.8)	63(14.9)	53(12.6)	
**Alcohol (N (%))**	yes	21(35.6)	8(13.6)	13(22)	5(8.5)	11(18.6)	1(1.7)	0.040
	no	103(21.9)	87(18.5)	117(24.9)	38(8.1)	65(13.8)	60(12.8)	
**Sleep duration** **median(min-max)**		8(4-14)	8(2-13)	8(2-12)	7(5-10)	7(4-10)	7(5-12)	0.006
**Hours of sitting** **median(min-max)**		8(2-20)	8(2-16)	8(1-15)	7(2-15)	6(2-16)	6(1-15)	0.009
**Distance to nearest sport club median(min-max)**		500(8-20000)	500(5-12000)	325(10-5000)	520(20-6000)	500(10-17000)	200(4-5000)	0.024
**Distance to nearest park median(min-max)**		500(6-10000)	400(5-10000)	200(5-5000)	500(30-2000)	350(5-5000)	200(4-3000)	0.380
**Fast food / processed food at month: median(min-max)**		2(0-25)	3.5(0-30)	2(0-30)	2(0-20)	3(0-50)	3(0-30)	0.650
**Useless Junk at month: median(min-max)**		2(0-31)	3(0-50)	3(0-100)	3(0-20)	2(0-25)	2(0-30)	0.110
**BMI (median)(mg/m^2^)**		24.39 (17.5-45.21)	23.60 (14.69-39.06)	23.52 (15.62-38.14)	23.44 (19.15-33.72)	24.12 (17.59-32.85)	23.53 (16.98-29.07)	0.310

To predict concerning factors related to physical activity behavior, logistic regression by backward stepwise method applied. Negative state of variables considered as reference. Age, gender, BMI, alcohol consumption, sleep duration, transporting by bicycle, having company and encouragement were identified as predictors of pre contemplation stage (Table 4). Gender, having compeer, transport by bicycle were predictors of termination phase (Table 5).

**Table 4:** Predictors of pre contemplation stage – logistic regression

	B	S.E.	Wald	df	p-value	OR
BMI	0.11	0.03	14.18	1	< 0.001	1.12
age	0.03	0.01	8.10	1	0.004	1.03
Gender (R*:women)	0.74	0.30	5.95	1	0.015	2.09
Distance to gym	< 0.001	< 0.001	3.10	1	0.08	1.00
compeer	-1.06	0.33	10.45	1	0.001	0.35
encouraging	0-.92	0.29	9.74	1	0.002	0.40
Sleep duration	0.24	0.09	7.21	1	0.007	1.28
bicycle	-20.28	16265.98	< 0.001	1	0.01	< 0.001
diabetes	-1.73	1.09	2.53	1	0.11	0.18
alcohol	0.89	0.40	5.01	1	0.02	2.44
Constant	-6.70	1.20	31.10	1	< 0.001	0.001

Variables entered on step 1: marital state, occupation, education, BMI, age, gender, family size, distance to park and gym, having compeer and encouraging, fast food and useless junk consumption, sitting position, sleep duration, transport by personal car, taxi, metro, bicycle and pedestrian, diabetes, respiratory and liver disease, CVD and cancer, smoking, hookah and alcohol. The negative state of variables considered as references. R square: 0.21

* =Reference

**Table 5:** Predictors of termination stage – logistic regression

	B	S.E.	Wald	df	p-value	OR
Gender (R*:women)	0.81	0.40	4.20	1	0.04	2.25
compeer	0.92	0.39	5.59	1	0.02	2.50
encouraging	0.81	0.45	3.23	1	0.07	2.25
Taxi	-1.92	1.04	3.41	1	0.06	0.15
bicycle	2.06	0.98	4.46	1	0.03	7.87
Respiratory disease	-19.19	10998.56	< 0.001	1	0.10	< 0.001
smoking	-0.92	0.52	3.10	1	0.08	0.40
alcohol	-1.74	1.05	2.77	1	0.10	0.17
Constant	-3.27	0.48	46.01	1	< 0.001	0.04

Variables entered on step 1, are the same as table 4. The negative states of variables considered as references. R square: 0.11

* =Reference.

## 4. Discussion

Regular physical activity contributes to a healthier lifestyle in all age groups and its positive effects on health are well documented (Emdadi, Nilsaze, Hosseini, & Sohrabi, 2007; Bouchard, Shephard, & Stephens, 1994). Despite public health efforts, the percentage of the people involved in healthful levels of physical activity is low (Centers for Disease Control and Prevention, 2005; Garber, Allsworth, Marcus, Hesser, & Lapane, 2008). To our knowledge, this study provides the first evidence about exercise patterns of Iranian population at community level, according to TTM change phases. All previous studies on this issue have been conducted on specific populations in Iran.

This study showed that a large number of people (more than half) were in the inactive stage (pre-contemplation, contemplation and preparation). These findings are consistent with other studies that most of the adolescents, students and adults in their research were in the early stages (Kim, 2007; Sharifirad, Charkazi, Tashi, Shahnazi, & Bahador, 2011; Dumith, Gigante, & Domingue, 2007). In Jordan and Courneya studies, however, most of the participants were in action and maintenance stages (Jordan, Nigg, Norman, Rossi, & Benisovich, 2002; Courneya & Bobick, 2000) which may be due to the fact that in developed countries regular physical activity is well organized and exercise equipment are more accessible. It is necessary to provide stage-matched intervention for improving physical activity and information about the benefits of exercise and risks of sedentary life for the individuals in earlier stages especially in pre-contemplation.

Our analysis showed that gender is a predictor of physical activity behavior. However, the relationship between the physical activity stages of change and gender was not similar to other studies (Moattari et al., 2013; Emdadi et al., 2007; Jordan et al., 2002; Irwin, 2004). In this study, women were more frequently in earlier stages and were among the least active participants. This can be due to cultural limitations and lack of adequate space and facilities for women in these communities. In our study, the number of men was higher than the number of women in all of the stages except for preparation. This may be because of the smaller proportion of women and so in regression analysis male gender was predictor for both pre-contemplation and termination phases. In one survey, there was not any difference between men and women regarding stages of change but that study only included vigorous activity (Boutelle, Jeffery, & French, 2004).

One of the predictors of pre-contemplation stage in this study was age and by increasing age people intended to be inactive. Most studies found that the proportions of older people are higher in the pre-contemplation stage and lower in the maintenance stage (Dumith, Gigante, & Domingue, 2007; Kearney, Graaf, Damkjaer, & Engstrom, 1999). However El-Gilany and Garber showed in his study that age was a non-significant independent variable for physical activity patterns. The effect of aging on physical activity patterns may be explained in part by increased level of chronic disease and disability in older adults (El-Gilany, Badawi, El-Khawaga, & Awadalla, 2011; Garber et al., 2008).

As BMI increased, participants were more likely to be in the pre-contemplation stage. Overweight individuals have been shown to be less physically active than normal weight subjects (Garber et al., 2008; Kearney et al., 1999). In Kearney research, the results for BMI differ from previous studies and showed individuals with higher BMI were more likely to present intention to begin, suggesting that being overweight may provide a motivation for a program of weight loss involving increased physical activity (Kearney et al., 1999).

We found that alcohol consumption had a significant relation with pre-contemplation stage and those drinking alcohol were more frequent in that stage but there was not any relation between smoking and this phase. Other studies found that the percentage of current smokers is higher in the pre-contemplation stage and lower in the maintenance stage than for the non-smokers or former smokers (Varo Cenarruzabeitia, 2003), but in Kearney study there was not any significant difference in numbers of smokers and alcoholics between pre-contemplation and maintenance stages (Kearney et al., 1999).

Having the support of family, encouraging and compeer for exercising was a key element in determining physical activity patterns. People with such support had the higher proportion in termination stage than pre-contemplation like another study (Boutelle et al., 2004); however, this finding was not consistent with Kearney survey (Kearney et al., 1999). Social environmental factors can help or hinder physical activity. The effect of these factors on physical activity habits includes the attitudes of family, peers, and health professionals. Support of the spouse and their attitude can be even more important than the participant’s (Dishman, Sallis, & Orenstein, 1985).

Relationship between sleep duration and physical activity was one of the other findings of this study. People with longer sleep duration had a higher percentage in earlier stages and it was found one of the predictors of pre-contemplation stage. Long sleep is associated with low physical activity, which is a strong predictor of death but the association between long sleep duration and death is not fully understood. Ballavia showed in their study that long sleep duration was associated with higher mortality risk and shorter survival only among participants with a low level of physical activity (Bellavia, Åkerstedt, Bottai, Wolk, & Orsini, 2014).

In this study, educational level, job, marital status, size of family, transportation mode and health status did not predict pre-contemplation and termination stages of exercise. This suggests that each group was as likely to begin an exercise initiation program and this is not limited to those with specific jobs and who can afford health clubs or those who are the member of a specific group of people such as different educational level, health status, and family size.

Extensive research has clearly revealed that people of different groups can promote their health by simply incorporating moderate levels of physical activity into their daily routine. Dishman expressed that the lack of progress toward the physical activity goals is a result of poor understanding of interventions and more research on what motivates individuals to adopt and maintain a physically active lifestyle is required (U.S. Department of Health and Human Services, 1996; Dishman, 1994). Extension exercise and health promotion interventions are likely to have a greater impact if they are tailored to the attributes and preferences of peoples. Most behavioral change research and behavior change interventions are designed for people in the decision stage (they are prepared for action) (Prochaska & Marcus, 1994). However, current research suggests that since a high percentage of people are in the pre-contemplation stage, like preparation stage, specific action directed towards earlier stages is needed. In an attempt to be more successful with interventions and promotion of physical activity, it is important to perform studies to recognize people’s difference in their motivation to become active and to tailor the counseling message according to the individual’s readiness for change.

Strengths of this study are as follows:

- To our knowledge, this is the first study to investigate the Stages of change for physical activity and related factors in Iranian adults. Most studies on this issue were performed for specific populations.
- The interviewers were trained for the questionnaires application and were not aware of the purpose of the study
- We did not have a random sampling but we do our best to have representative samples by collecting them throughout the city.

It is important to keep the following limitations in mind: first; interpretation of the relationship between the physical activity stages of change and the determinants should match the study design and because of cross-sectional design that all variables were measured simultaneously, their association does not necessarily establish causation. Second; data are subjective and self-reported, actual physical activity patterns was not measured in our study. It is possible that the self-reporting nature of physical activity in this research allow over-reporting of exercising. Third; influence of seasonality on exercise patterns is another issue to be considered. The level and the motivation to involve in exercise tend to be higher in spring and summer, the time that our survey was done. The comparison of the results is difficult sometimes, because definition of physical activity and stages of change is different in some studies. Longitudinal designs are recommended in order to examine the stability of different physical activity predictors across time and remove the seasonality effect.

## 5. Conclusion

The results of this study indicated that the majority of the people are in the sedentary stages and do not meet the recommended levels of exercise behavior. According to established benefits of regular physical activity, it is necessary to tailor intervention to enhance the physical activity among individuals. Factors identified as predictors of physical inactivity such as age, sex, BMI and family and friend support should be taken into account in the design of interventions.

Bases on TTM research for sedentary, subjects move from inactive stages to active stages with emphasizing on the personal, long-term benefits of physical activity, a decrease in the barriers and cons of exercise will be useful in facilitating adoption exercise.
